# Filamentous fungi for sustainable remediation of pharmaceutical compounds, heavy metal and oil hydrocarbons

**DOI:** 10.3389/fbioe.2023.1106973

**Published:** 2023-02-14

**Authors:** Soumya Ghosh, Iryna Rusyn, Olena V. Dmytruk, Kostyantyn V. Dmytruk, Helen Onyeaka, Marieka Gryzenhout, Yusufjon Gafforov

**Affiliations:** ^1^ Department of Genetics, Faculty of Natural and Agricultural Sciences, University of the Free State, Bloemfontein, South Africa; ^2^ Department of Ecology and Sustainaible Environmental Management, Viacheslav Chornovil Institute of Sustainable Development, Lviv Polytechnic National University, Lviv, Ukraine; ^3^ Institute of Cell Biology NAS of Ukraine, Lviv, Ukraine; ^4^ Institute of Biology and Biotechnology, University of Rzeszow, Rzeszow, Poland; ^5^ School of Chemical Engineering, University of Birmingham, Birmingham, United Kingdom; ^6^ Mycology Laboratory, Institute of Botany, Academy of Sciences of Republic of Uzbekistan, Tashkent, Uzbekistan; ^7^ AKFA University, Tashkent, Uzbekistan

**Keywords:** bioremediation, removal efficiency, pharmaceutical compounds, heavy metals -, oil hydrocarbons, pollutants

## Abstract

This review presents a comprehensive summary of the latest research in the field of bioremediation with filamentous fungi. The main focus is on the issue of recent progress in remediation of pharmaceutical compounds, heavy metal treatment and oil hydrocarbons mycoremediation that are usually insufficiently represented in other reviews. It encompasses a variety of cellular mechanisms involved in bioremediation used by filamentous fungi, including bio-adsorption, bio-surfactant production, bio-mineralization, bio-precipitation, as well as extracellular and intracellular enzymatic processes*.* Processes for wastewater treatment accomplished through physical, biological, and chemical processes are briefly described. The species diversity of filamentous fungi used in pollutant removal, including widely studied species of *Aspergillus*, *Penicillium*, *Fusarium*, *Verticillium*, *Phanerochaete* and other species of Basidiomycota and Zygomycota are summarized. The removal efficiency of filamentous fungi and time of elimination of a wide variety of pollutant compounds and their easy handling make them excellent tools for the bioremediation of emerging contaminants. Various types of beneficial byproducts made by filamentous fungi, such as raw material for feed and food production, chitosan, ethanol, lignocellulolytic enzymes, organic acids, as well as nanoparticles, are discussed. Finally, challenges faced, future prospects, and how innovative technologies can be used to further exploit and enhance the abilities of fungi in wastewater remediation, are mentioned.

## Graphical Abstract

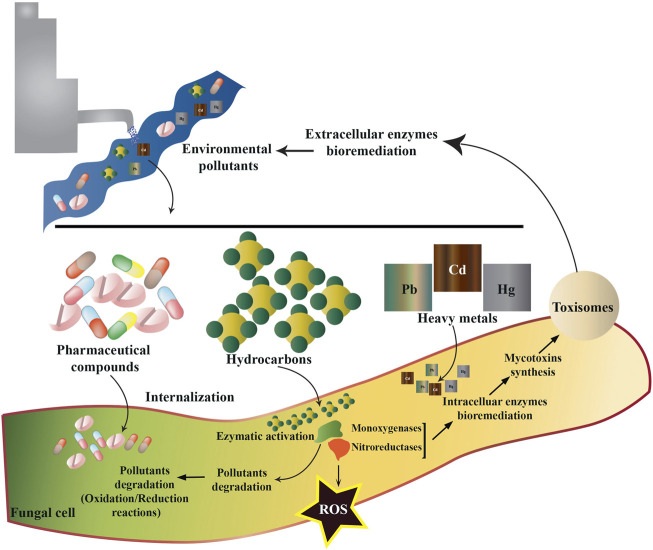

## 1 Introduction: History and benefits of using filamentous fungi for wastewater treatment

The microbial world are well-known for the vast diversity of functionalities they present (Dunlap, 2001; Shu and Huang, 2022). These functionalities have been exploited by humans to produce novel products (Stahl et al., 2013; Vuong et al., 2022), but also to degrade human produced waste (Raziyafathima et al., 2016; Chiang et al., 2020; Gupta et al., 2022). Miraculously microbes have always been discovered that could degrade most of the novel products that humans synthesized and that become waste. This is besides those that can already degrade naturally occurring organic compounds that occur in higher concentrations than normal due to human activities. Surprisingly these attributes of microbes are still, relatively speaking, vastly underutilized in industry and rehabilitation activities, despite a number of success stories that have already been applied (Gupta et al., 2022; Rafeeq et al., 2022). The same holds true for the industrial application of microbes that forms the topic of this review, namely the sustainable rehabilitation of specifically wastewater, which is a very complex field.

Fungi are, similar to other microbes such as bacteria and algae, incredibly useful in bio-industries (Dhiman et al., 2022; Hadibarata et al., 2022). Numerous applications using fungi exist, including several in the field of wastewater rehabilitation. In fact, in some cases fungi can be synergistically used together with other microbes, and not just on their own (Gultom and Hu, 2013; Chu et al., 2021). Their abilities stem from the fact that they form the foundation of every ecosystem on Earth, and that they occupy every possible niche and geographical area where they are saprobes, parasites or symbionts (Chaudhary V. B. et al., 2022b; Tedersoo et al., 2022). In order to survive, they thus need a wide range of enzymatic abilities, such as pectinases, cellulases and phosphatases, and can produce diverse types of compounds, such as siderophores (Haripriyan et al., 2022). Their ability to withstand a range of environmental conditions, of which some are extreme similar to those in waste waters, makes them highly beneficial. However, ironically the majority of applications in waste water rehabilitation uses algae and bacteria.

The biodiversity of fungi that has thus far been used in waste water rehabilitation is staggering. The fungi used encompasses various phyla of the fungi, and within phyla, they are also diverse (Bhunjun et al., 2022). The ease by which fungi can be grown in fermentation, selected from nature or where known cultures from culture collections can be tested for their ability to degrade or convert a specific compound, gives them incredible potential to be used in green technology in an eco-friendly manner (Yang and Peng, 2022). This is especially so when native fungi are exploited with possible better or novel properties. Often, fungi can be isolated from environments mimicking waste areas, or the target environment itself, because they are already present, for example as sediment fungi, metallophyles or fungi from extreme environments (Chaudhary P. et al., 2022a; Talukdar et al., 2022). They can then be made into a product that can be easily added to the problem area.

Compounds that must be degraded, or converted, in waste waters for rehabilitation are challenging because they are so incredibly diverse. These compounds range from metals and other elements that are not supposed to be present in specific environments, various types of oils such as crude oils, derived products, and food based oils, and biocides. Human made products such as plastics, varnish, foams, dyes, disinfectants, hygiene products and pharmaceuticals, add further to this list of xenobiotics (Gultom and Hu, 2013; Chu et al., 2021). Due to the saprobic nature of fungi, they are also useful where too high concentrations of organic matter, such as cattle farms with manure, crop based agricultural wastes, fisheries or abattoirs, are found (Camenzind et al., 2022; Ediriweera et al., 2022). Moreover, often beneficial by-products can be developed by the fungi from these problem areas (Guo et al., 2022).

This review presents a comprehensive summary of the latest research that has been mentioned above in waste water bioremediation utilizing specifically fungi. It covers cellular mechanisms, processes for wastewater treatment, a summary of the fungi involved and how they are utilized for the various types of pollutants, and beneficial byproducts made by fungi. Lastly, challenges faced, future prospects, and how innovative technologies can be used to further exploit and enhance the abilities of fungi in wastewater remediation, are mentioned.

## 2 Mycoremediation mechanisms

### 2.1 Non-enzymatic processes

**FIGURE 1:**
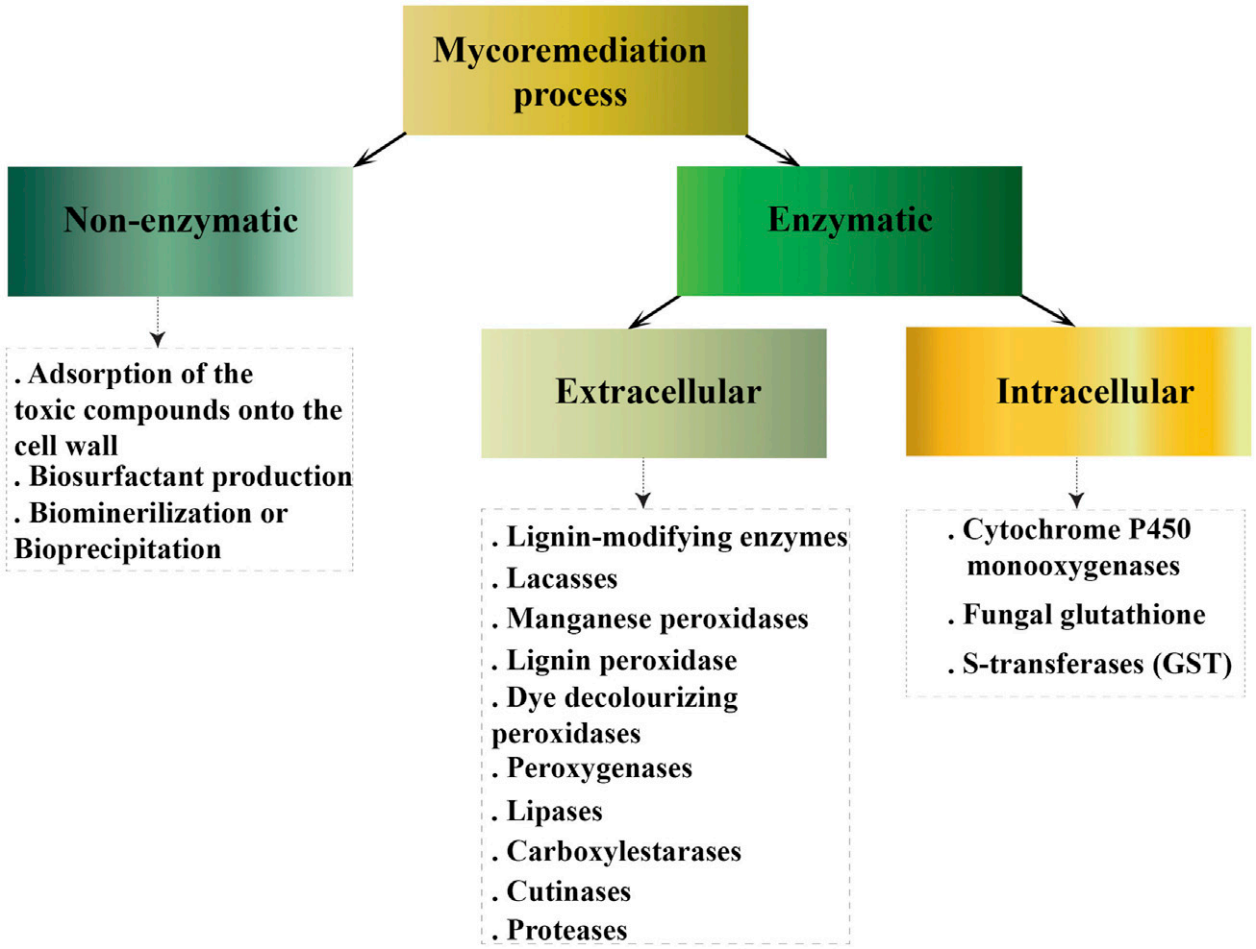
Cellular mechanisms involve in mycoremediation.

There are several mechanisms of bioremediation described for filamentous fungi (Figure 1). The first one is adsorption of the toxic compounds into the cell wall. Bioadsorption is a physico-chemical process in which the concentration of toxic compounds is adsorbed on the bio-surface of fungi. Absorption takes place *via* specific aminopolysaccharides that are part of the composition of the cell wall, such as chitosan or chitin (Kumari et al., 2016). The functional groups of glucosamine in chitosan act as metal absorbent sites for heavy metal chelation or complexation facilitating the reduction and recovery of heavy metal from the polluted environment (Alsharari et al., 2018; Geetha et al., 2021). Absorption is the predominant mechanism of bioremediation of the heavy metals Copper (Cu) and Cobalt (Co.) described for *Trichoderma*, *Penicillium*, and *Aspergillus* (Dusengemungu et al., 2020). In filamentous fungi *Phoma* sp. biosorption into fungal mycelia has an important role for some pharmaceutical compounds removal, until this bioadsorption reaches equilibrium (Hofmann and Schlosser, 2016). Filamentous fungi *Mucor* shows considerable promise to be applied as a mycoremediation agent for the removal of cyanobacterial toxins from aquatic environments due to excellent toxin uptake into the fungal cell (Balsano et al., 2015). Removal of Fe^3+^ and Co^2+^ by fungi *Aspergillus* sp. AHM69 and *Penicillium* sp. AHM96 was shown *via* biosorption and bioaccumulation on the biomass surface (El-Bondkly and El-Gendy, 2022)*.*

The other mechanism of bioremediation is associated with biosurfactants production by filamentous fungi. These biomolecules reduce the surface/interfacial tension between fluid phases (da Silva et al., 2021). Lipopeptides, molecules consisting of a short amino acid chain, covalently modified with lipids are well known class of biosurfactants, proved suitable for *bioremediation* of crude oil (Carolina et al., 2021). The biotechnological potential of *Penicillium*, *Aspergillus* and *Fusarium* for Lipopeptides production is described (Gautam et al., 2014; Qazi et al., 2014; Bills et al., 2016). The produced crude biosurfactant or fungal strain *Mucor circinelloides* could be directly used for the mycoremediation of crude oil contamination in oil fields (Hasanizadeh et al., 2017). However, microbial biosurfactant production is still quite restricted, since the production costs are currently higher than chemical surfactants. Besides most bioprocesses show low yield and productivity.

The other mechanism of filamentous fungi to counteract with a toxic compounds is biomineralization or bio-precipitation. Oxalate is a key metabolite that plays a significant role in many metal and mineral transformations mediated by fungi. Oxalic acid, (COOH)_2_ is a relatively strong organic di-acid with the ability of the oxalate anion to complex metals including those of Co, Cd, Cu, Mn, Mg, Ni, Sr, Pb and Zn (Gadd, 1999). It is generally stated that metal oxalate formation may confer protection from the potentially toxic effects of such metals (Johansson et al., 2008; Gadd et al., 2014). Oxalic acid produced by a variety of filamentous fungi (Gadd et al., 2014).

It is well described that in *Aspergillus niger,* oxalic acid synthesized by conversion of oxaloacetate to oxalate and acetate by a cytoplasmic oxaloacetate acetylhydrolyase. Oxaloacetate in turn synthesized from pyruvate, coming from glycolysis, by the action of pyruvate carboxylase (Pedersen et al., 2000a; Pedersen et al., 2000b). This pathway may be widely distributed in fungi (Gadd et al., 2014). Oxalate is potentially toxic in high concentration and needs to efflux from cells. It was demonstrated copper oxalates formation by *A*. *niger* (Frank-Kamenetskaya et al., 2021) that can be used to develop of *copper bioremediation* from soil. A strain of *Aspergillus nomius*, isolated from a heavily-polluted soil from a zinc smelting region, was able to precipitate zinc oxalate (Sutjaritvorakul et al., 2016).

Lead is one of the most widely found toxic metal contaminants in the environment arising from industrial activities (Flora et al., 2012). Complete removal of Pb from solution and extensive precipitation of lead oxalate (PbC_2_O_4_) around the mycelium of *A*. *niger* and *Paecilomyces javanicus* was demonstrated (Liang et al., 2016). Other study also demonstrated reduction of Pb toxicity by *Aspergillus niger* and *Penicillium oxalicum via* formation of insoluble Pb minerals, primarily lead oxalate (Tian et al., 2019). *Penicillium chrysogenum* was effectively utilized for the biomineralization of chromate and lead from an aqueous solution and contaminated soil. It was shown efficient fungal-mediated calcite precipitation of heavy metals in the remediation (Qian et al., 2017).

### 2.2 Enzymatic processes

The above mentioned mechanisms include non-enzymatic process. The other strategies include biotransformation and biodegradation mediated by enzymatic systems. Enzymatic fungal-mediated biotransformation in turn, can be carried out by means of extracellular and intracellular enzymes. Extracellular enzymes catalyze variety of reactions (e.g., oxidative coupling products, hydroxylation, CO_2_ emission, ether cleavage, aromatic ring fission, quinoid products formation and other) resulting in biotransformation of toxic compounds facilitating bioremediation processes (Singh et al., 2021). The biodegradation is particularly committed for filamentous fungi since they can produce the extracellular non-specific lignin-modifying enzymes, laccases, manganese peroxidases, lignin peroxidase, dye decolorizing peroxidases, peroxygenases (El-Gendi et al., 2022).

Decolourization is a process in which filamentous fungi are particularly effective (Al-Ansari et al., 2022; Dhir, 2022). This process involves the removal of color from a solution, often by breaking down the colored compounds present in the solution. Filamentous fungi have been shown to accomplish this through the production of laccase and manganese peroxidase enzymes (Bilal et al., 2016a; Bilal et al., 2016b). Laccase is a type of enzyme that can break down a wide range of colored compounds through oxidation, acting on a variety of colored compounds such as azo dyes and natural pigments (Srinivasan and Sadasivam, 2018). Therefore, fungal laccases were widely applied for the bioremediation of complex synthetic dyes by altering the dye molecules into non-colored, safer, and ecofriendly structures (Sanghi et al., 2009; El-Fakharany et al., 2016). Manganese peroxidase (MnP) is another enzyme that can be involved in decolorization by filamentous fungi. MnP uses hydrogen peroxide as a cofactor to oxidize a wide range of compounds, particularly lignin-derived compounds which are found in industrial effluents and are responsible for the color of the solution (Bilal et al., 2016b). The use of filamentous fungi in bioremediation processes can take place in different types of reactors, including solid-state fermentation, submerged fermentation, or adsorption-biodegradation (Svobodová and Novotný, 2018; Zapana-Huarache et al., 2020; Usuldin et al., 2021). The process can be improved by optimizing the environmental conditions, such as pH, temperature, and oxygen availability, and by selecting the appropriate fungal strains. It is also important to note that decolorization alone may not remove other pollutants in the solution, thus the process should be combined with other remediation techniques, such as biodegradation, in order to achieve complete remediation of the contaminated site (Bilal et al., 2016a; Bilal et al., 2016b).

On the other hand, polycyclic aromatic hydrocarbons are abundant and widespread contaminants for the environment. *Aspergillus oryzae* and *Mucor irregularis* isolated from crude oil efficiently degraded hydrocarbons from oil by the action of secreted laccase, manganese peroxidase, and lignin peroxidase (Asemoloye et al., 2020). Plastic pollution poses serious environmental problems, in part due to the extremely stable and durable nature of this polymer. Fungal secreted hydrolases (lipases, carboxylesterases, cutinases and proteases) able to modify the plastic surface, increasing its hydrophilicity (Vertommen et al., 2005). These enzymes were successfully used to degrade polyethylene terephthalate and polyurethane hydrolyzing chemical bonds in the polymer structures (Cregut et al., 2013; Webb et al., 2013). Highly stable carbon–carbon bonds in plastic polymers require oxidation before the depolymerisation process (Restrepo-Florez et al., 2014). These reactions efficiently facilitate secreted oxidoreductases (laccases and peroxidases) which are involved in plastic degradation into smaller molecules such as oligomers, dimers and monomers (Alvarez-Barragan et al., 2016). Fungal laccases and peroxidases are very promising enzymes in degrading polyethylene and polyvinyl chloride (Temporiti et al., 2022). Secreted fungal laccases and peroxidases enzymes can be effectively used for detoxification of heavy metals and pharmaceuticals (Olicon-Hernandez et al., 2017; Mousavi et al., 2021).

Intracellular enzymes participating in bioremediation of toxic compounds can be divided in two groups. First–cytochromes and second–glutathione transferases. The cytochrome P450 system is a large family of enzymes, mainly monooxygenases catalyzing reactions of hydroxylation, oxygenation, dealkylation, epoxidation of C=C bonds, reduction, and dehalogenation (Díaz-Cruz et al., 2014). It was shown that P450 play an indirect but crucial role in the process of clearance of cadmium and lead in filamentous fungi *Phanerochaete chrysosporium* belonging to the Basidiomycota phylum (Zhang et al., 2015). CYP63A2 P450 monooxygenase from this fungus oxidized polycyclic aromatic hydrocarbons, alkylphenols, and alkanes from crude oil (Syed et al., 2013). Cytosolic and mitochondrial iso-forms of P450 from *Fusarium oxysporum* and other fungi are used in degradation of dioxins (Guengerich and Munro, 2013; Sakaki et al., 2013). *F. oxysporum* P450 monooxygenases are promising catalysts in production of omega hydroxy fatty acids when heterologously expressed in *Saccharomyces cerevisiae* (Durairaj et al., 2015). It was demonstrated that several *Aspergillus* species are capable of degrading a carcinogenic contaminant Benzo [*a*]pyrene by the action of the cytochrome P450 monooxygenase (Loss et al., 2019).

Fungal glutathione S-transferases (GST) are detoxification enzymes which can catalyze the conjugation of glutathione to non-polar compounds containing an electrophilic carbon, nitrogen, or sulfur atom. By this mechanism, glutathione S-transferases are able to metabolize drugs, pesticides, xenobiotics, heavy metals and other toxic compaunds (Pocsi et al., 2004). GST have been reported to contribute to the defense against heavy metal stress in different filamentous fungi including *A*. *nidulans* (Fraser et al., 2002). Glutathione transferases play an important role in the intracellular reduction of Cr (VI) in fungi (Garcia-Hernandez et al., 2017). It was shown that the detoxification of penicillin side-chain precursors might depend on microsomal GST in *P*. *chrysogenum* (Emri et al., 2003). Fungal cytochromes can catalase a lot of different reactions, which enables them to degrade xenobiotics and pollutants (Syed et al., 2013; Chen et al., 2019; Wang et al., 2019; Carstens et al., 2020; Hu et al., 2020). Fungal enzymes, isolated from Basidiomycetes and Ascomycetes, show high ability of plastic waste biodegradation. Fungal laccases and peroxidases, generally used by fungi to degrade lignin, polyethylene and polyvinyl chloride, while esterases such as cutinases and lipases were successfully used to degrade polyethylene terephthalate and polyurethane (Temporiti et al., 2022).

## 3 Processes involved in wastewater treatment

Wastewater treatment necessitates the expenditure of energy, time, and money. Any solution that can enhance the wastewater treatment process is critical since one of the sustainable development goals is to minimise energy consumption and strategic processes globally (Shojaei and Shojaei, 2021). Treatment of wastewater could be accomplished through physical, biological, and chemical processes.

### 3.1 Physical wastewater treatment process

Physical wastewater treatment process refers to separating particle matter and solids in sanitary and industrial effluents (Pirzadeh, 2022). Depending on the sewage type, fabric fragments, sand, plastic bits, and tree foliage may be in the wastewater entering the treatment plants. These particles can cause damage to the equipment (including pipes, pumps, and fittings) used in wastewater treatment. Furthermore, failing to remove these things puts a lot of strain on the machinery in biological and chemical wastewater treatment, lowering the output quality.

In the physical wastewater treatment process, mechanical procedures based on physical rules are employed to remove pollutants (Woodard, 2001). Physical operations are typically simpler and more quantitatively effective than other wastewater treatment methods. Physical wastewater treatment techniques remove pollutants using naturally occurring forces, including electrostatic attraction, gravity, van der Waal forces, and physical barriers (Tripathi and Misra, 2019). In general, the techniques involved in physical treatment do not lead in changes in chemical composition of the target compounds. In some cases, the physical state is altered, as in vaporization, and scattered substances are often made to agglomerate, as in filtration (Crittenden et al., 2012). The first phase in every wastewater treatment system is filtration (Gerba and Pepper, 2019). This procedure requires the separation of non-biodegradable contaminants from a wastewater treatment plant. Sedimentation, skimming, adsorption, aeration, and flotation are physical methods of wastewater treatment (Saravanathamizhan and Perarasu, 2021), as are barriers such as deep bed filters, screens, bar racks, and membranes.

### 3.2 Chemical wastewater treatment process

Chemical wastewater treatment is another process of wastewater treatment. It is considered a tertiary treatment process that incorporates chemical treatment (Ahmed et al., 2021). Chemicals are used in this process, which involves separating or converting pollutants because of their chemical reaction. Chemical precipitation, neutralization, disinfection (ozone, chlorine, UV light), ion exchange, and adsorption are the most commonly used chemical treatment processes (Samer, 2015).

### 3.3 Biological wastewater treatment

Biological wastewater treatment involves the use of microorganisms in wastewater treatment, a poorly understood process that combines biology and biochemistry (Sahu et al., 2019). This process is used in removing organic, soluble, biodegradable, organic and nutrient-containing compounds, and colloids, present in wastewater. The wastewater is introduced into a specifically designed bioreactor, where organic matter is utilised by bacteria (anaerobically or aerobically), fungi and algae (aerobically) (Samer, 2015). The bioreactor provides suitable environmental conditions for microorganisms to grow and utilize dissolved organic materials as a source of energy. The biological oxidation of organic substances will continue as long as the microorganisms receive oxygen and food from collected wastewater. Bacteria, which form the fundamental trophic level of the food chain in the bioreactor, carry out the majority of the biological process.

The conversion of dissolved organic molecules into thick bacterial biomass *via* bioconversion has the potential to purify wastewater (Meena et al., 2021). Following that, sedimentation is used to segregate the microbial biomass from the treated wastewater. Microorganisms breakdown organic materials *via* two distinct biological processes: biosynthesis and biological oxidation (Samer, 2015). Biosynthesis converts colloidal and dissolved organic materials into dense biomass, which then can be recovered *via* sedimentation. In this case colloidal and dissolved organic molecules are converted into dense biomass, which can then be removed *via* sedimentation. Some biological oxidation by-products, such as minerals, remain in the solution and are eliminated with the effluent.

## 4 Species diversity of filamentous fungi used in pollutant removal

Filamentous fungi are organism belonging to a large group of eukaryotes that includes some yeasts, moulds and some mushrooms. Filamentous fungi, like fungi in general, belong to a separate kingdom. They are unable to photosynthesis, possess urea metabolism and glycogen storage. Fungi reveal the predominance of the osmotrophic nutrition over the phagotrophic and reproduce using spores. The main difference between fungi and other organisms is the peculiarity of the structure of the cell wall containing chitin. However, key characteristic contributing to classifying fungi as filamentous fungi is the ability to possess hyphae. These hyphae, in turn, form branches making up fungal mycelia, growing like threadlike structures.

Filamentous fungi are phylogenetically diverse; however, members of three groups, namely Ascomycetes, Basidiomycetes, and Zygomycetes are mostly found in association with bioremediation research studies, or commercial exploitation (Troiano et al., 2020).

A huge number of known filamentous fungi belong to the phylum Ascomycota (Moretti, 2009). Filamentous fungi encompass many genera with *Aspergillus, Penicillium, Fusarium, Verticillium* and some other more investigated than the other genera for their suitability of use in bioremediation (Olicon-Hernandez et al., 2017) (Table 1).

**TABLE 1:** Species diversity of filamentous fungi used in pollutant removal.

Pollutants	Species	References
	Ascomycota	
*Cd*	*Aspergillus niger*	Barros et al. (2003)
	*A. clavatus* DESM	Cernansky et al. (2007)
*Cu,Pb,As*	*A. niger*	Clausen (2004), Mukherjee et al. (2010)
*Cr*	*A. niger, A. foetidus*	Prasenjit and Sumathi (2005), Srivastava and Thakur (2006)
Ni, Pb bioleaching	*A. niger*	Le et al. (2006), Chakraborty et al. (2014)
Crude oil	*A. ramosus, A. flavus*	((Davies and Westlake, 1979)
Gasoline	*A. oryzae*	(Binsadiq, 1996; Oboh et al., 2006)
*PAH*	*A. niger, A. terreus*	Capotorti et al. (2004), Okoro, 2008; Ali et al. (2012)
Dye	*Aspergillus spp.*	Devi and Kaushik (2005), Ponraj et al. (2011)
Acidic and basic dyes	*A. foetidus, A. niger*	Ryu and Weon (1992), Sumathi and Manju (2000), Fu and Viraraghavan (2002)
*Cd*	*Penicillium simplicissimum*	Fan et al. (2008)
	*P. chrysogenum*	Niu et al. (1993), Holan and Volesky (1995), Skowronski et al. (2001)
	*P. canescens*	Say et al. (2003)
*Zn*	*P. digitatum*	Galun et al. (1987)
	*P. simplicissimum*	Fan et al. (2008)
	*P. chrysogenum*	Niu et al. (1993)
	*P. spinulosum*	Townsley and Ross (1985)
*Pb*	*P. simplicissimum*	((Fan et al., 2008)
	*P. chrysogenum*	Niu et al. (1993)
	*P. canescens*	Say et al. (2003)
Cu	*P. chrysogenum*	Skowronski et al. (2001)
	*P. cyclopium*	Ianis et al. (2006)
	*P. spinulosum*	Townsley and Ross (1985)
	*P. italicum*	Mendil et al. (2008)
As, Hg, Cr	*P. canescens*	Say et al. (2003)
Mn, Fe, Ni, Co.	*P. italicum*	Mendil et al. (2008)
Fluorene	*P. canescens*, *P. janczewskii*, *P. montanense*, *P. simplicissimum*, *P. restrictum*	Garon et al. (2004)
	*P. chrysogenum*, *P. italicum*	Garon et al. (2002)
Pyrene	*P. harzianum*, *P. terrestre*	Saraswathy and Hallberg (2002)
	*P. ochrochloron*	Saraswathy and Hallberg (2005)
	*P. chrysogenum*, *P. aurantiogriseum*, *P. crustosum*, *P. rugulosum*	Ravelet et al. (2000)
Phenol	*P. simplicissimum*	Marr et al. (1996)
	*P. chrysogenum*	Leitao et al. (2007)
2-, 3- and 4-Chlorophenol	*P. frequentans* Bi 7/2, *P. simplicissimum* and *P. simplicissimum*	Hofrichter et al. (1994), Marr et al. (1996)
Olive mill wastewater	*P. decumbens*	Borja et al. (1992)
Vinasses	*Penicillium* spp.	Jimenez et al. (2005)
Coffee residues	*P. curstosum*, *P. verrucosum*, *P. crustosum*, *P. restrictum*, *P. implicatum*, *P. citrinum*	Fujii and Takeshi (2007)
Tl	*Fusarium* sp. FP, *Arthrinium* sp. FB, and *Phoma* sp. FR	Mo et al. (2022)
PAH	*Fusarium* sp. ZH-H2	Zhao et al. (2019)
Chlorpyrifos	*Verticillium* sp	Farhan et al. (2021)
Pb, Zn	*V. insectorum* J3	Feng et al. (2018)
	Basidiomycota	
Anthracene	*Phanerochaete chrysosporium*	Vyas et al. (1994), Novotny et al. (2004)
Naphthalene	*Ph. chrysosporium*	Mollea et al. (2005)
Phenanthrene	*Ph. chrysosporium*	Bumpus (1989)
Pyrene	*Ph. chrysosporium*	Novotny et al. (2004)
Pentachlorophenol	*Ph. chrysosporium*	Bumpus (1989), Lamar and Dietrich, 1990 (1992)
DTT, lindane	*Ph. chrysosporium*	Bumpus (1989)
2,4,6-Trinitrotoluene	*Ph. chrysosporium*	Fernando et al. (1990)
PAH	*Xerotus discolor*	Acevedo et al. (2011)
	*Bjerkandera specie*	Matsubara et al. (2006)
	*Irpex lacteus*	Bhatt et al. (2002), Cajthaml et al. (2006)
	*Phellinus* sp.	Arun and Eyini (2011)
	*Schizophyllum commune*	Matsubara et al. (2006)
	*Stropharia coronilla*	Steffen (2000)
Pentachlorophenol	*Agrocybe perfecta*, *T. villosa*, *T. hirsuta*	Machado et al. (2005)
	*Ceriporiopsis subvermispora*	(Lamar and Dietrich, 1990; 1992)
AH	*Allescheriella* sp.	D'Annibale et al. (2006), D'Annibale et al. (2007)
Nitrobenzene, anthracene	*Coriolopsis polyzona*	Vyas et al. (1994)
	*C. trogii*	Levin et al. (2003)
	Zygomycota	
Diazinon	Cunninghamella elegans ATCC36112	Zhao et al. (2020)
DiclofenacR	*Mucor hiemalis*	Esterhuizen-Londt et al. (2017)
Malachite greenR	*Cunninghamella elegans*R	Cha et al. (2001)
CopperR	*Mortierella* spp.	Fu et al. (2022)
PyreneR	*Mucor mucedo*	Hou et al. (2015)
PAHsR	*Мucor* spp.R	Gupta et al. (2015)
Crude oilR	*Мucor* spp.R	Okoro et al. (2013)

The genus of *Aspergillus* consists of different species applied in bioremediation of virtually all types of environmental hazards. This genus is of particular interest due to its rich species variety, with various ecological functions. *Aspergillus* contain some unique enzymes, rarely produced by other microorganism, some of them help the species to counteract toxic substances and some are responsible for bioremediation of a wide range of toxic substances.

A lot of strains of *Aspergillus* work as excellent biosorbents for heavy metals removal, including cadmium from oil field water (Barros et al., 2003) and from aqueous solution (Cernansky et al., 2007) copper, lead, arsenic (Clausen, 2004; Mukherjee et al., 2010), chromium (Prasenjit and Sumathi, 2005; Srivastava and Thakur, 2006). *A. niger* is using to produce a variety of organic acids for the leaching of heavy metals from contaminated soils (Wasay et al., 1998; Ren et al., 2009) and is effective in the bioleaching of nickel laterite ores and Pb (Le et al., 2006; Chakraborty et al., 2014). Dead biomass of *Aspergillus* can be used as an effective biosorbent of heavy metals (Rostami and Joodaki, 2002). Pretreated and modification of the fungal cell surface increased the sorption of heavy metals (Kapoor et al., 1999; Rao et al., 2005). Strains of *Aspergillus* are capable of degrading hydrocarbons, such as crude oil (*Aspergillus ramosus*) (Davies and Westlake, 1979), gasoline (Binsadiq, 1996; Oboh et al., 2006), polycyclic aromatic hydrocarbons (Capotorti et al., 2004; Okoro, 2008; Ali et al., 2012), crude oil (Adegunlola et al., 2012). Some of the *Aspergillus* sp. are well adapted to wastewater cleaning (Devi and Kaushik, 2005; Ponraj et al., 2011) and are able to decolorizing acidic and basic dyes (Ryu and Weon, 1992; Sumathi and Manju, 2000; Fu and Viraraghavan, 2002).

*Penicillium* spp. are very important for the processes of environmental remediation. They can work as a naturally biosorbents for heavy metal environmental reduction. Some strains of *Penicillium* [*P. simplicissimum* (Fan et al., 2008), *P. chrysogenum* (Niu et al., 1993; Holan and Volesky, 1995; Skowronski et al., 2001)], *P. canescens* (Say et al., 2003) are able to remediate cadmium. A lot of strains can absorb zinc [*P. digitatum* (Galun et al., 1987), *P. simplicissimum* (Fan et al., 2008), *P. chrysogenum* (Niu et al., 1993), *P. spinulosum* (Townsley and Ross, 1985)], Pb [*P. simplicissimum* (Fan et al., 2008), *P. chrysogenum* (Niu et al., 1993), *P. canescens* (Say et al., 2003)], Cu [*P. chrysogenum* (Skowronski et al., 2001), *P. cyclopium* (Ianis et al., 2006), *P. spinulosum* (Townsley and Ross, 1985), *P. italicum* (Mendil et al., 2008)], As, Hg, Cr [*P. canescens* (Say et al., 2003)], Mn, Fe, Ni, Co. (*P. italicum*) (Mendil et al., 2008).

Polycyclic aromatic hydrocarbons comprise a large group of organic compounds with two or more fused aromatic rings, that occur naturally in coal, crude oil, and gasoline. A lot of Penicillium spp can remediate these chemicals. Fluorene is an ortho-fused tricyclic hydrocarbon that is a major component of fossil fuels and their derivatives. This chemical can be remediated by *P. canescens*, *P. janczewskii*, *P. montanense*, *P. simplicissimum*, *P. restrictum* (Garon et al., 2004), *P. chrysogenum*, *P. italicum* (Garon et al., 2002). Pyrene is a polycyclic aromatic hydrocarbon consisting of four fused benzene rings, resulting in a flat aromatic system. It can be degraded by *P. harzianum*, *P. terrestre* (Saraswathy and Hallberg, 2002), *P. ochrochloron* (Saraswathy and Hallberg, 2005), *P. chrysogenum*, *P. aurantiogriseum*, *P. crustosum*, *P. rugulosum* (Ravelet et al., 2000). *Such* polycyclic aromatic hydrocarbons like benz [a]pyrene, phenanthrene, fluoranthene also can be utilized by fungi (Sack and Gunther, 1993).

Several Penicillium strains have the ability to transform phenol and its toxic derivatives into products that are less mutagenic. For example, *P. simplicissimum* (Marr et al., 1996) and *P. chrysogenum* (Leitao et al., 2007) can degrade phenol compounds. 2-, 3- and 4-Chlorophenol can be metabolized by *P. frequentans* Bi 7/2, *P. simplicissimum* and *P. simplicissimum* (Hofrichter et al., 1994; Marr et al., 1996).

*P. decumbens* have high catabolic enzyme activity and can utilize a lot of simple aromatic compounds, including detoxification of olive mill wastewater (Borja et al., 1992). *Penicillius* also take part in the remediation of vinasses (Jimenez et al., 2005) and coffee residues (*P. curstosum, P. verrucosum, P. crustosum, P. restrictum, P. implicatum, P. citrinum*) (Fujii and Takeshi, 2007) in liquid wastes.

Different *Fusarium* strains are adapted to higher concentrations of heavy metals (Co, Cu, and Mn) and can be used as natural biosorbents for bioremediation (Poonam et al., 2016). *Fusarium* sp. FP, *Arthrinium* sp. FB, and *Phoma* sp. FR can remove Thallium Tl (I) from aqueous solutions (Mo et al., 2022). Combination of *Fusarium* sp. ZH-H2 and starch offers a suitable alternative for bioremediation of aged polycyclic aromatic hydrocarbon (PAH)-contaminated soil in coal mining areas (Zhao et al., 2019).

Fungi Verticillium sp. exhibited the successful degradation of pesticides, such as chlorpyrifos in different mediums (Farhan et al., 2021). *Verticillium insectorum* J3 have efficient biosorption mechanism for Pb (II) and Zn (II) and can be used for the development of biotreatment technologies for heavy metal-polluted waste (Feng et al., 2018).

The phylum Basidiomycota include filamentous fungi with enormous bioremediation potential. *Phanerochaete chrysosporium* is perhaps the most widely used basidiomycete species for bioremediation. A wide spectrum of hydrocarbons can be bioremediated by *Phanerochaete chrysosporium* thanks to their various enzyme systems (Paszczynski and Crawford, 1995; Cameron et al., 2000). These fungi are able to degrade anthracene (Vyas et al., 1994; Novotny et al., 2004), naphthalene (Mollea et al., 2005), phenanthrene (Bumpus, 1989), pyrene (Novotny et al., 2004) and pentachlorophenol (Bumpus, 1989; Lamar and Dietrich, 1990; 1992). Such pollutants as DTT, lindane (Bumpus, 1989) and 2,4,6-Trinitrotoluene (Fernando et al., 1990) can also be completely degraded by *P. chrysosporium.* A high level of polycyclic aromatic hydrocarbon biodegradation was reported for such fungi as *Xerotus discolor* (Acevedo et al., 2011), *Bjerkandera species* (Matsubara et al., 2006), *Irpex lacteus* (Bhatt et al., 2002; Cajthaml et al., 2006), *Phellinus sp*. (Arun and Eyini, 2011), *Schizophyllum commune Fr* (Matsubara et al., 2006), *Stropharia coronilla* (Steffen, 2000). Pentachlorophenol can be degraded by *Agrocybe perfecta*, *T. villosa (Sw.)* (Machado et al., 2005), *T. hirsuta* and *Ceriporiopsis subvermispora* (Lamar and Dietrich, 1990; 1992), aromatic hydrocarbons (AH) can be remediated by *Allescheriella sp.* (D'Annibale et al., 2006; D'Annibale et al., 2007), nitrobenzene and anthracene are reported to be degraded by *Coriolopsis polyzona* (Vyas et al., 1994) and *C. trogii* (Levin et al., 2003).

Some well-studied species of filamentous fungi representatives of Zygomycota are used for bioremediation (Olicon-Hernandez et al., 2017). *Cunninghamella elegans* have been extensively used as model fungi in different studies on the metabolism of xenobiotics (Asha and Vidyavathi, 2009). *Cunnninghamella* spp., have the ability to degrade insecticides and dyes (Cha et al., 2001; Zhao et al., 2020). It was shown that *Cunninghamella elegans* ATCC36112 could effectively degrade the pesticide (diazinon) mediated by cytochrome P450 (Zhao et al., 2020). Another genus of the fungus Zygomycota, *Mortierella*, obviosly сould considered as additional bioremediation agent of cooper contaminated soil (Fu et al., 2022). Content of hydrocarbons of a crude oil and PAHs was decreased with *Mucor* spp., (Okoro et al., 2013; Gupta et al., 2015). The degradation of pyrene and polycyclic aromatic hydrocarbons (PAHs) residues was shown by *Mucor mucedo* immobilizing on carrier-corncob of contaminated agricultural soil (Hou et al., 2015). *M. hiemalis* decrease concentrations of diclofenac in the media (Esterhuizen-Londt et al., 2017). Thus, filamentous fungi of all groups Ascomycota, Basidiomycota, and Zygomycota have a good remedial potential and can be suitable for removing from polluted environments both pharmaceutical compounds and heavy metals, as well as oil hydrocarbons and other pollutants.

## 5 Recent progress in bioremediation by filamentous fungi

Anthropogenic activity leads to increasing pollution of the environment with a rising set of organic and inorganic compounds, deteriorating the state of the ecosystem. Filamentous fungi with a wide range of extracellular and intracellular enzymes, surfactant production and, biosorption properties and the ability to symbiosis can change the approaches to bioremediation and wastewater treatment. Long remediation time and slow growth serves as limit factors the application mycoremediation, however, recent research offers an opportunity to overcome these challenges.

### 5.1 Wastewater treatment of pharmaceutical compounds

The excessive amount of pharmaceutical compounds released into environments poses a long-term risk to humans and wildlife, even under low concentration (Angeles et al., 2020; Akerman-Sanchez and Rojas-Jimenez, 2021). Large range of common pharmaceutical compounds were found in wastewater treatment plants and effluent waters, including analgesics, antidiabetics, anti-inflammatories, antibiotics, anti-hypertensives, beta-blockers, diuretics, and lipid regulators negativelly. These compounds negatively impact on environmental and aquatic ecosystems health (Olicon-Hernandez et al., 2019; Akerman-Sanchez and Rojas-Jimenez, 2021). Filamentous fungi belong to the microorganisms responsible for the degradation of most organic compounds polluting environment (Bulkan et al., 2020; Ferreira et al., 2020). Increasing studies indicate that filamentous fungi can transform many of pharmaceutical compounds, including antibiotics, anti-inflammatories, β-blockers, etc., (Bulkan et al., 2020; Alamo et al., 2021; Kasonga et al., 2022). Recent studies have shown prospects of biotechnology based on filamentous fungi for almost complete elimination of a wide range of pharmaceutical compounds that may have ecotoxic effects in the environment (Table 2). Reports (Dalecka et al., 2020a; Dhiman et al., 2022) have shown that filamentous fungi can efficiently remove pharmaceutical compounds at level of 78%–100% from a synthetic wastewater media. Research of past years mostly have shown a slow rate of degradation of pharmaceutical compounds with a treatment duration of more than 26 days (Li et al., 2015; Badia-Fabregat et al., 2016; Mir-Tutusaus et al., 2017). However, studies with the white-rot fungus *Trametes versicolor* (Shreve et al., 2016) and recent results with species of *Aspergillus, Mucor, Penicillium, Rhizopus, Trametes* and *Trichoderma* demonstrate (Mohapatra et al., 2022; Shourie and Vijayalakshmi, 2022) demonstrated significant degradation with high removal rates in 4 h–10 days (Olicon-Hernandez et al., 2019; Dalecka et al., 2020b; Alamo et al., 2021; Kasonga et al., 2022). Using adsorption to solid supports, specifically, polyurethane foam carriers for immobilized white rot fungus *Trametes versicolor* resulted in 99.9% diclofenac removal after even 4 h during batch experiments (Stenholm et al., 2019). The choice of carrier and the level of cell immobilization are importance in this process. Whereas, experiments that utilized polyethylene-carriers with negligible immobilization of *T. versicolor*, a 98% total diclofenac removal was achieved only after one week investigation. To optimize the elimination of pharmaceuticals, the type of bioreactor acts as another important factors. The fungal fixed-bed reactor could be more suitable than the stirred tank reactor to remove pharmaceutical compounds from wastewater, since it was able to remove PhACs to a great extent and simultaneously detoxify real wastewater. A trickle-bed bioreactor (TBB) using fungal biomass immobilized on rice husks, achieved an elimination of 88.6% and 89.8% in synthetic and real wastewater, respectively (Tormo-Budowski et al., 2021). Additionally, toxicological tests showed a decrease in the hospital wastewater’s toxicity after the treatment in the TBB. The exploring with focusing on optimizing the conditions inside the bioreactors reveals as meangfull for the effective mycoremediation process (Akhtar and Mannan, 2020). A novel idea with effective and fast treatment of actual pharmaceutical wastewater containing β-lactam antibiotics was presented in recent research (Ji et al., 2021). *Aspergillus niger* mycelial pellets were used as biological carriers for displaying β-lactamase on the cell surface of fungi. This *A. niger*-Bla system significantly improved the removal of antibiotics for > 60% and demonstrated complete degradation of amoxicillin and ampicillin within only 1 h, and cefamezin at 80.45%.

**TABLE 2:** Pharmaceutical compounds (PhCs) removal efficiency by filamentous fungi.

Strain	Pollutant	Removal efficiency %	Time	References
*Aspergillus luchuensis*	Diclofenac	> 99.9%	10 days	Dalecka et al. (2020b)
*Aspergillus niger*	diclofenac	78–100	24 h	Kasonga et al. (2022)
*Mucor circinelloides*	diclofenac	78–100	24 h	Kasonga et al. (2022)
	ibuprofen	98	2 days	
*Penicillium oxalicum*	diclofenac	99	24 h	Olicon-Hernandez et al. (2019)
*Rhizopus microspores*	carbamazepine	87	10 days	Kasonga et al. (2022)
	diclofenac	78–100	24 h	
*Rhizopus* sp.	carbamazepine	100	9 days	Kasonga et al. (2022)
	diclofenac	100		
	ibuprofen	100		
*Trametes versicolor*	45–79 of different PhCs	69	–	Alamo et al. (2021)
	16 of different PhCscarbamazepine	88.6–89.8	14 days	Tormo-Budowski et al. (2021)
	trimethoprim	34–82	48 h	Alharbi et al. (2019)
	sulfamethoxazole	39–95	48 h	Stenholm et al. (2019)
	diclofenac	49–56	48 h	
		98–99	4 h - 1 week	
*Trametes polyzona*	diclofenac	78–100	24 h	Kasonga et al. (2022)
*Trichoderma longibrachiatum*	diclofenac	78–100	24 h	Kasonga et al. (2022)

Degradation of individual pharmaceuticals was more efficient than their elimination from а mixture. For example, diclofenac (5 mg/L) was completely removed by *Trametes versicolor* to below its detection limit (1 mg/L) within 8 h in the individual experiment vs. after 24 h in a mixture with the other pharmaceuticals (Alharbi et al., 2019). A similar trend was visible with trimethoprim (TMP), carbamazepine (CBZ), and sulfamethoxazole (SMX), with 95% vs. 39%, 82% vs. 34%, and 56% vs. 49% removal after 48 h with 5 mg/L of TMP, CBZ, and SMX individually or as mixtures, respectively.

Thе ability of filamentous fungi to degrade a wide variety of pharmaceutical compounds and their easy handling make them excellent tool for the bioremediation of emerging contaminants of pharmaceutical origin. The study of different carriers, such as solid ones for fungal cells or biological ones for their enzymes, type of fungal reactor serves as important role that can accelerate mycoremediation time and removal effeciency of pharmaceutical compounds and bring closer the time of their large-scale application for wastewater treatment.

### 5.2 Heavy metal treatment

Filamentous fungi can be considered as an important factor in bioremediation and heavy metal treatment due to their ability of biosorption, bioacumulation and metal recovery. Filamentous fungi capable to tolerating high metal concentrations, can decontaminate the environment encumbered with heavy metal pollutants (Manguilimotan and Bitacura, 2018; Kapahi and Sachdeva, 2019; Dusengemungu et al., 2021). Filamentous fungy can be used in recovery of many essential metals from various metal sources, including solid mine wastes and polluted soils, mine tailings and electronic waste, spent catalysts and low-grade ores solubilizing metals through the secretion of organic acids (Ghosh et al., 2020; Dusengemungu et al., 2021) (Table 3).

**TABLE 3:** Heavy metal removal efficiency by filamentous fungi reported recently.

Strain	Metal	Removal efficiency %	Waste	References
*Aspergillus carbonarius*	Al	70–80	Spent Catalyst	Das et al. (2019)
*Aspergillus foetidus*	Al	88.43	Spent Catalyst	Das et al. (2019)
*Aspergillus fumigatus*	Cd	79.0	Polluted soil	Khan et al. (2014)
*Aspergillus nidulans mutant*	Cu	70.0	Industrial effluents	Rissoni Toledo et al. (2021)
*Aspergillus niger*	Al	80–81	Spent Catalyst	Das et al. (2019)
*Aspergillus niger* and *Penicillium simplicissium*	Al	≥ 98	Bauxite	Shah et al. (2020)
*Aspergillus niger*	Cd	98	Polluted soil	Khan et al. (2014)
*Aspergillus niger*	U	80	Uranium mine	Sun et al. (2020a)
*Aspergillus niger*	Pb	98.4	Aqueous Solution	Nuban et al. (2021)
	Pb	100	Sewage	Ghyadh et al. (2019)
*Aspergillus niger*	Cr	100		Xu et al. (2021)
*Aspergillus tubingensis*	Pb	90.8	Polluted soil	Tang et al. (2021)
	Zn	68.4		
	Cr	64.5		
*Cladosporium* sp. A	Mg	74	Mine tailings	Huerta-Rosas et al. (2020)
	Ag	67		
*Cladosporium* sp. B	Mg	58	Mine tailings	Huerta-Rosas et al. (2020)
	Ag	40		
*Mucor hiemalis*	Al, Cd, Co., Cr, Cu, Hg, Ni, Pb	> 81%–99%	Polluted water	Hoque and Fritscher (2019)
	U, Zn			
	Ag, Au, Ti			
*Penicilium chrysogenum*	Mg	69	Mine tailings	Huerta-Rosas et al. (2020)
	Ag	53		
*Penicillium simplicissimum*	Cu	90	Waste electrical equipment	Arshadi et al. (2019)
	Ni	89		
*Penicillium rubens*	Cd	98	Polluted soil	Khan et al. (2014)
*Rhizopus oligosporium*	Cd	65	Sewage	Ghyadh et al. (2019)

One of the most studied in this regard is the genus *Aspergillus* which demonstrates high leaching and removal capacity, as well as multi-metal tolerant properties. The removal capacity of *A. niger* was reached at 100% from Cd and Pb polluted sewage in 6 days and only 65% was reached when using *Rhizopus oligosporium* (Ghyadh et al., 2019). *Aspergillus* species are known for their ability to tolerate different metals such as Al, Cd, Co, Cr, Cu, Fe, Mn, Mg, Ni, Pb, Zn, and U (Dusengemungu et al., 2021; Naveen Kumar and Prakash, 2021). A unique microbial biotechnology for simultaneous bioremediation and biomining of twelve ionic metals overcoming the obstacles of multimetal toxicity to microbes were described. Three new strains of *Mucor hiemalis* was demonsrated multimetal-resistance, hyper-accumulation and elicitation power, intracellular fixing and deposition of mercury as nanospheres in sporangiospores (Hoque and Fritscher, 2019). Microbiomes, germinated spores and dead insoluble cellwalls of these strains removed > 81%–99% of applied Al, Cd, Co, Cr, Cu, Hg, Ni, Pb, U, and Zn simultaneously and furthermore enriched precious Ag, Au and Ti from water all within 48 h (Hoque and Fritscher, 2019).

The eco-friendliness and cost-effectiveness of recent advances in metal biorecovery and bioremediation by fungi show that filamentous fungi have an exceptional impact for industrial-scale bioremediations in future operations. Problems of optimization of metal recovery technology, how to extract metals from the cells after bioaccumulation/biosorption, as well as exploration of optimal alternative sources of energy for fungal growth are challenges for the current research community, that will be necessary to overcome for commercialization fungal bioremediation.

### 5.3 Oil hydrocarbons bioremediation

The use of microorganisms for bioremediation and restoration of oil hydrocarbons contaminated soils and aquatic ecosystems have the challenge that a huge amount of petroleum hydrocarbons accumulated in environments that are of great toxicity (Varjani, 2017; Chicca et al., 2022). Bioremediation of crude oil polluted environments is often limited by the scarcity microorganisms with complementary substrate specificity required for degrading different hydrocarbons (Varjani, 2017; Anno et al., 2021; Chicca et al., 2022). Filamentous fungi are reported for their ability to degrade hydrocarbon pollutants, similar to many bacteria (Widdel and Rabus, 2001; Xu et al., 2018; Zahri et al., 2021), yeasts (Rusyn et al., 2003; Farag and Soliman, 2011; Al-Dhabaan, 2021) and algae (Al-Hussieny et al., 2020; Anno et al., 2021; Ghodrati et al., 2021). Depending on the strain of filamentous fungi characterized the rate of crude oil removal efficiencies ranged from 30.4% to 98.1% during 7–10 days (Table 4). Consortia of filamentous fungi and bacteria e.g. *Aspergillus* and *Bacillus*, was more effective in bioremediation and reached 99.0% in comparison with monocultures which utilized 98.0% and 20.0% in 7 days (Perera et al., 2021). The ability of *Penicillium* sp. to tolerate oil pollutants from 2.5% to 10% and degradate more than 50% suggest that it could be employed as an bioremediation agent for restoring the ecosystem when contaminated by oil (Vanishree et al., 2014; Dirisu et al., 2018). Among 84 filamentous fungal isolates belonging to eight different genera, *Penicillium polonicum* AMF16, *P. chrysogenum* AMF47 and two isolates affiliated to *P. cyclopium*, were determined as the most promising isolates for bioremediation of crude oil pollution (1%–5%) in the marine environment within the frame of bioaugmentation or biostimulation processes (Maamar et al., 2020). Degradation of petroleum hydrocarbons both by single filament strains and in synergism with bacteria allows progressive increases in bioremediation of contaminated sites.

**TABLE 4:** Bioremediation of crude oil and hydrocarbons by filamentous fungi reported recently.

Strain	Pollutant	Removal efficiency %	Time	References
*Aspergillus* sp. RFC- 1	crude oil (1%)	60.3	7 days	Al-Hawash et al. (2019)
	naphthalene	97.4		
	phenanthrene	84.9		
	pyrene	90.7		
*Aspergillus ustus* HM3	crude oil (2%)	30.4	10 days	Benguenab and Chibani (2021)
*Aspergillus* sp. MM1 and *Bacillus* sp*.* MM1	crude oil (8 g/L)	99.0	7 days	Perera et al. (2021)
*Mucor* sp	crude oil	65.0	3 weeks	Dirisu et al. (2018)
*Penicilium* sp	crude oil	55.0	3 weeks	Dirisu et al. (2018)
*Purpureocillium lilacinum* HM4	crude oil (2%)	44.5	10 days	Benguenab and Chibani (2021)

## 6 Fungal byproduct recovery

### 6.1 Traditional fungal byproducts

Wastewaters containing pollutants such as pharmaceutically active compounds, heavy metals, aliphatic and polycyclic aromatic hydrocarbons, herbicides, pesticides, surfactants, dyes, personal care products and others can be purified by filamentous fungi (Ferreira et al., 2020; Morsi et al., 2020). Waste or side streams generated from the agricultural, forest, industrial, food and municipal sectors can be converted to value-added components by these organisms. Side streams are generally rich in carbohydrates (lignocellulose, starch, sugar), proteins, fat, minerals, etc., that can be converted to a range of value-added components such as fungal biomass, ethanol, hydrolytic enzymes, and organic acids.

Wastewaters, in contrast to side streams, contain minor amounts of carbohydrates and nitrogen sources commonly used by filamentous fungi. Thus, for removal of pollutants, additional nutrients should be spent to support growth of filamentous fungi. As a result, fungal biomass and enzymes are only obtained value-added products. The use of fungal biomass extracts as nutrients can be applied to enrich wastewaters or side streams where nutrient supplementation is required (Nair et al., 2017). It is necessary to classify side streams aimed for bioremediation, focused on removal of pollutants, and those aimed for valorization into products, for integration into anthropogenic activities. Some strategies were proposed for integration of valorization of side streams with removal of pollutants from wastewaters by filamentous fungi. For example, paper and pulp industries produce side streams that have been considered in both perspectives (Ferreira et al., 2020). However, there are no commercial processes presently known.

Filamentous fungal biomass is the main value-added component. It is generated independently of the intended valuable product to be produced. Fungal biomass grown in different side streams contains 40%–60% protein, as well as profiles of amino acids and polyunsaturated fatty acids similar to those in fishmeal and soybean meal, which are the main protein sources for animal feed. Several representatives of filamentous fungi, e.g., *A. oryzae, Fusarium venenatum, Monascus purpureus, Neurospora intermedia, Rhizopus microsporas, Rhizopus oligosporus,* and *Rhizopus oryzae* have been used for production of fermented foods, and are recognized as GRAS (Generally recognized as safe) microorganisms (Ferreira et al., 2020; Sar et al., 2022b). Their GRAS status is a necessary point for consideration of fungal biomass for feed applications. Filamentous fungi have been used for biomass production on a variety of food industry side-streams such as fish industry wastewaters, vinasse, olive oil mill wastewater, thin stillage, etc., (Ferreira et al., 2015; Karimi et al., 2019; Sar et al., 2020a; Sar et al., 2020b; Sar et al., 2021). It was described that fungal biomass production by Rhizopus delemar at an industrial scale contained 53% crude protein from edible potato protein liquor (Sar et al., 2022b). The R. delemar fungal biomass can be a promising raw material for feed and food production, since its protein and fatty acid profiles include 41% essential amino acids and 33% polyunsaturated fatty acids (Sar et al., 2022a).

Despite the fact that fungal biomass first of all is considered as a promising material for feed and food production, other potential applications of residual fungal biomass are proposed. The cell wall of fungal biomass contain chitosan, chitin, and β-glucans, with confirmed immune-stimulation activities when used in feed recipes (Karimi et al., 2018). Additionally, potential applications of chitin and chitosan in pharma, cosmetics, bioplastics, biopolymers, and agricultural sectors are well described (Isaza-Perez et al., 2020).

The filamentous fungus *Mucor indicus* provided promising results in heavy metal removal from wastewaters (Javanbakht et al., 2011). The biomass of this fungus has shown a great potential to be used as a rich nutritional source. Cells of *Mucor indicus* are great sources of chitosan and polyunsaturated fatty acids particularly γ-linolenic acid (Karimi and Zamani, 2013). Moreover, this fungus produced high ethanol production levels during fermentation of glucose and lignocellulosic hydrolysates, which is an important value-added product. Increase of ethanol yield from glucose was reached by supplementation with fungal biomass autolysates (Asachi et al., 2011).

*Aspergillus oryzae* and *Neurospora intermedia* were employed for the production of ethanol and high-protein biomass by cultivation on enzymatically liquefied bread-waste medium (Kawa-Rygielska et al., 2022). The cultivation of *Neurospora intermedia* in wheat-derived thin stillage resulted in extra ethanol production (Ferreira et al., 2015). *Fusarium oxysporum* is among the few filamentous fungi able to ethanol production directly from lignocellulose biomass. This fungus revealed the ability to produce ethanol from *Ficus* fruits employing a mild hydrothermal pretreatment without supplementing any extraneous enzymes using (Nongthombam et al., 2022). A cellulolytic thermophilic filamentous fungus *Myceliophthora thermophila* revealed huge potential for ethanol production from glucose and cellobiose. Recombinant strains expressing the cellodextrin transport system from *N. crassa* and alcohol dehydrogenase from *Saccharomyces cerevisiae* produced increased amounts of ethanol from cellobiose during fermentation at 48°C (Li et al., 2020).

Filamentous fungi play a significant role in the production of a wide range of lignocellulolytic enzymes, which are important value-added products (Ghosh, 2015; Ghosh et al., 2021). Such hydrolytic and oxidative enzymes are used for the production of important metabolites. Wild type strains have limited efficiency in enzyme production. Despite great progress in optimizing the cultivation conditions for enzyme production (Ferreira et al., 2016), molecular engineering methods were used for more pronounced improvement of the fungal production of lignocellulolytic enzymes. Constitutive production of pectinases (Alazi et al., 2019) and arabinolytic enzymes (Reijngoud et al., 2019) in *Aspergillus niger* were reached by point mutation in GaaR and AraR regulators, respectively. Deletion of the negative regulator CreA resulted in higher production of hemicellulase (Robl et al., 2018) in *A. niger*. Overexpression of mutated AraR in *Penicillium oxalicum* lead to constitutive production of α-L-arabinofuranosidase (Gao et al., 2019). Deletion of the regulatory gene Atf1 increased cellulase and xylanase production in *P. oxalicum* (Zhao et al., 2019). Deletion of repressors SxlR and Rce1 in *Trichoderma reesei* significantly stimulated xylanase (Liu et al., 2017) and cellulase (Cao et al., 2017) activities, respectively. Constitutive and increased production of xylanases and cellulases was also reached using the recombinant *Trichoderma reesei* overexpressing an artificial transcription activator Xyr1-Cre1b (Zhang et al., 2017).

Organic acids, predominantly citric, gluconic and itaconic acids, are value-added compounds produced by filamentous fungi (Ferreira et al., 2016). Citric acid production is the second largest fermentation product after ethanol production (Dhillon et al., 2013). Citric acid is mostly used in the food industry. Currently, *A. niger* is used for large scale production of this acid from sucrose or glucose containing substrates such as molasses or glucose syrup (Tsay and To, 1987; Show et al., 2015). To decrease citric acid production costs, other cost-effective substrates were investigated, e.g. apple pomace, orange processing waste, starch, rape seed oil (Behera, 2020). Huge efforts were made to optimize the fermentation process (Barrington et al., 2009). A significant increase of citric acid production was reached with metabolic engineering approaches of *A. niger* directed to carbon utilization and respiratory chain improvement, precursor biosynthesis enhancement, by-product removal and feedback inhibition reduction improvement (Tong et al., 2019).

Gluconic acid is an important biotechnological product used in food, feed, beverage, textile, pharmaceutical and construction industries. Gluconic acid produced with *A. niger from glucose can be done at an industrial scale* (Ramachandran et al., 2006; Yan et al., 2022). Other representatives of *Aspergillus* and *Penicillium* spp. have also been recognized as robust producers of this acid (Ma et al., 2022). Environmental and economical friendly processes of gluconic acid production trended to replace those using glucose and sucrose form waste products (e.g., hydrolysates of sugarcane, corn stover, corn cob, tea waste, starch, inulin, whey, figs, bananas, grapes, surpluses, wastepaper and lignocellulose) (Ferreira et al., 2016).

Itaconic acid is widely used in chemical synthesis industries (Wierckx et al., 2020). It was reported that itaconic acid suitable for the synthesis of antimicrobial biopolymers, drug carriers, intelligent food packaging, superabsorbent polymers, hydrogels in water treatment and analysis (Teleky and Vodnar, 2021). Itaconic acid production is carried out by fermentation more commonly using *Aspergillus* terreus from molasses or glucose (Huang et al., 2021). But currently several filamentous fungi are genetically engineered to produce this acid in high quantities and on different bio-wastes substrates (Sun L. et al., 2020b). Overall, filamentous fungi have great potential for further applications as robust producers of value-added products.

### 6.2 Mycosynthesis of nanoparticles

Furthermore, fungal biomass grown in heavy metal-rich media can be used as a source of nanocatalysts and nanoparticles (NPs) with potential application in chemical industries and micropollutant removal approaches (Huang et al., 2018; Kratosova et al., 2019). Mycosynthesis of metal-containing nanoparticles is alternative to conventional physico-chemical processes (Sebesta et al., 2022). This is a innovative biological approach to nanoparticle synthesis which leads its beginning from 2001 (Mukherjee et al., 2001). Then, formation of silver and gold nanoparticles using the fungus *Verticillium* was shown below the cell wall surface due to reduction of the metal ions by enzymes present in the cell wall membrane. NPs can be produced extracellular, outside the cell walls, which makes their separation from the biomass a simple process (Yadav et al., 2015; Priyadarshini et al., 2021). Some NPs synthesize intracellular, on the cell walls and even on the inner side of the cell walls (Mukherjee et al., 2001; Priyadarshini et al., 2021). Currently, nanoparticles of a wide range of different chemical compounds are known which are synthesised with the help of а filamentous fungi, such as metal Ag, Au, Cu, Pb, Pt, and bimetallic Ag-Au NPs, and also oxides, such as BaTiO3, Bi2O3, CoFe2O4, Co3O4, Fe2O3, Fe3O4, NiO, TiO2, ZnO, and ZrO2 (Mousa et al., 2021; Priyadarshini et al., 2021; Sebesta et al., 2022). Myconanoparticles can be used in various fields, i.e., in biomedicine, antimicrobial applications, catalysis, biosensing, mosquito control, and precision agriculture (Sebesta et al., 2022).

## 7 Problems associated with the fungal treatment systems and future prospects

Microscopic examination of hundreds of biomass samples from industrial effluent treatment plants and municipal wastewater treatment plants have revealed different operational issues. One of the main disadvantages of fungal reactors is that their wastewater treatment is not cost-effective (Espinosa-Ortiz et al., 2016). Others include filamentous bulking, toxicity, and dispersed growth, while fungal scattered mycelium typically causes bioreactor operation problems, including foaming, higher mixing and oxygen supply requirements, and growth on reactor walls and agitators.

Currently, various technologies are used to determine the toxicological mechanism of action of mycotoxins of these filamentous fungi. Among them, molecular biology techniques allow understanding the basic structure of nucleic acids and other molecules and help mimic their natural functions in both *in vivo* and *in vitro* studies. They are used to assess various aspects of cytotoxicity, cellular responses, gene expression, and the activation of specific signaling pathways and transcription factors (Manzoni et al., 2018). Notably, the use of zebrafish for *in vivo* toxicological research has been rapidly expanding (de Castro et al., 2016; Della Torre et al., 2018; Parenti et al., 2019; Taufek et al., 2020; Wan-Mohtar et al., 2020; Wan-Mohtar et al., 2021). Due to their easy bred, reared, easy laboratory maintenance, stability and ease of stable genetic manipulations, zebrafish are positioned as an ideal vertebrate model for *in vivo* studies compared to other vertebrates (Tonelli et al., 2020). For example, a toxicity study of exopolysaccharides, obtained from medicinal mushroom mycelial extracts, conducted on zebra fish embryo has been used as safety screening approach prior to pre-clinical testing and considered as national and international standards (Usuldin et al., 2021).

Fungi are not typically found in substantial amounts in aerobic wastewater treatment systems, but given the correct set of growth or environmental condition, fungi can grow out of control, negatively affecting treatments and effluent quality (Mir-Tutusaus et al., 2018). Fungi, on average, require a longer hydraulic retention time to remove pollutants from wastewater than bacteria. This complicates incorporating a fungal treatment step into a normal wastewater treatment system (Mir-Tutusaus et al., 2018). Some wastewaters cannot be treated with fungi. For example, fungal wastewater treatment systems are unsuitable for anoxic treatment of groundwater where oxygen is lacking. Fungi require aerobic conditions to grow and carry out other cellular activities (Trueba-Santiso et al., 2017). In addition, fungal treatment systems need additional nutrients, even while organic micro pollutants include carbon, some fungi require an extra assimilable carbon source for survival and growth (Mir-Tutusaus et al., 2018).

Bacteria outperform fungi and not just in natural settings but also in bioreactors. When compared to fungi, bacteria can survive a wider range of environmental conditions, proliferate faster, and destroy a wider range of contaminants (Espinosa-Ortiz et al., 2016). Another problem associated with the fungal treatment systems is in controlling bacterial contamination, which is one of the most common issues in the non-aseptic fungal treatment of wastewater (Espinosa-Ortiz et al., 2016). The proliferation of bacteria during fungal treatment of wastewater causes strong competition for the limited organic substrate, which impacts fungal metabolism because bacterial growth occurs on fungal filaments as support media (Badia-Fabregat et al., 2017; Hu et al., 2022). In addition, in industrial applications, wastewater sterilization is quite expensive. As a result, sustaining fungal biomass dominance in wastewater necessitates developing technologies to boost fungal competitive advantages over the bacteria microflora, while inhibiting growth of bacteria.

Fungal-bacterial mix for pollutant removal in fungal-pelleted bioreactors has been found in some situations to even improve the breakdown of some pollutants. As a result, further research is needed in the creation of fungal-bacterial symbiosis consortia for wastewater treatment. Additionally, more research is needed to expand the practicability of fungi in wastewater treatment, evaluate the economic, environmental, and technical aspects of fungal biofilm technologies in removing recalcitrant chemicals, and build a fungal biofilm immobilization technology for wastewater treatment.

## 8 Conclusion

The research that has been done up to now, and the applications that could be developed, indicate that only the surface of using fungi in sustainable wastewater remediation has been scratched. Research has only been done in a relatively limited number of geographical locations compared to other regions of the globe, and only a small percentage of fungi of the number of species out there, have been tested or exploited. Actually, most likely candidates for rehabilitation of specific problems were selected from a limited pool. Numerous opportunities exist, including the production of revenue in the form of byproducts contributes to the sustainability of these fungal applications, especially for a circular bio-economy model. A better understanding of the genes involved in these properties of used fungi, further opens up opportunities, enhances understanding, could solve some of the current problems experienced, and opens up recombinant DNA solutions. The ease by which fungi can be integrated in nanoparticle applications also enhances their abilities. It is thus likely that with the correct mindset solutions to the current problems faced in this field can be found in future.
